# Building transformative capacity for adaptation planning and implementation that works for the urban poor: Insights from South Africa

**DOI:** 10.1007/s13280-018-1141-9

**Published:** 2019-02-08

**Authors:** Gina Ziervogel

**Affiliations:** Department of Environmental and Geographical Science, and African Climate and Development Initiative, University of Cape Town, Cape Town, 7700 South Africa

**Keywords:** Adaptation planning, Inclusive governance, Local government, South Africa, Transformative capacity, Urban risk

## Abstract

The intersecting challenges of urbanization, growing inequality, climate and environmental risk and economic sustainability require new modes of urban governance. Although the urban poor are increasingly recognized as needing to be part of climate adaptation planning and implementation, many governance arrangements fail to explicitly include them. In order to make climate governance more inclusive, transformative capacity is needed. Drawing on two case studies from different urban contexts in South Africa, this paper explores the nature of inclusive governance between local government and the urban poor and the extent to which this has contributed to transformative development trajectories. The findings suggest that inclusive governance will be strengthened when local government (1) recognizes the everyday reality of the urban poor and works with them to identify priorities for transformative change, (2) supports sustained intermediaries who are urban poor themselves and (3) draws on diverse modes of governance to find new ways to engage diverse actors and experiment with inclusive adaptation planning and practice. These practices will help to build transformative capacity that can envisage and enable new ways of governing urban risk and implementing adaptation that puts the poor, frequently most impacted by climate and disaster risk, at the centre.

## Introduction

Multi-lateral agreements such as the Urban SDG, New Urban Agenda, Paris agreement and others are increasingly seeing cities as well placed to contribute to achieving ambitious targets (Revi et al. 2014; Parnell 2016; Solecki et al. 2018). Within the climate change context, this is particularly acute given the recognition of the role of cities to limit warming to 1.5 degrees, as well as being central to adapting to and reducing climate risk (de Coninck 2018; Solecki et al. 2018). The persistent failure of cities to adequately address equity and social inclusion has prompted calls for inclusive urbanization, seeing local governments as catalysts or principal agents in social transformation, particularly in the context of climate change (Shrestha et al. 2014; McGranahan et al. 2016; Amundsen et al. 2018; Solecki et al. 2018). On the other hand, some authors argue that there is a limit to what urban governments might be expected to achieve, particularly in cities that are under-capacitated and unrepresentative (Parnell et al. 2007; Satterthwaite 2011).

In order to shift current trajectories more rapidly towards the possibility of meeting the SDGs and associated goals and targets, the call for urban transformation to systematically address environmental and specifically climate risk alongside social change has been embraced (Revi et al. 2014; Solecki et al. 2018; Bai et al. 2018). The concept of urban transformation builds on diverse theoretical origins, but commonly takes a systems perspective and acknowledges the need for change across social, institutional, ecological and physical dimensions (Wolfram et al. 2016; Amundsen et al. 2018; Romero-Lankao et al. 2018). In the context of climate change, Pelling et al. (2015) argue that adaptive actions that shift development pathways can be seen as transformative. Drawing on social ecological systems and political ecology literature, Solecki et al. (2018, p. 38) define urban transformation, as “fundamental change in the system configuration… deemed necessary and implemented, putting the core of formerly established system configurations into question”. O’Brien (2018) suggests that this systemic change is needed across three spheres of transformation, namely the practical (behavior and technical responses), political (systems and structures) and personal (beliefs, values and worldviews) dimensions of transformation.

At the heart of achieving many of these ambitious global targets, in the context of cities, is the emphasis on shifting to a pro-poor approach, across research, practice and policy (IPCC 2014; Pieterse et al. 2015; Brown and McGranahan 2016; Parnell 2016; Castán Broto 2017). As McGranahan et al. (2016) suggest, in the context of urbanization, this requires a shift from the historic focus on economic growth, which has been exclusionary, towards reframing the city as inclusionary. They argue that “rather than becoming easier to deal with over time, exclusion can leave a toxic social legacy of socio-spatial inequality, segregation and compromised formal authority, and rising violence” (McGranahan et al. 2016, p. 21). They go on to argue that if urban inclusion can be achieved, cities will be more likely to achieve human rights and broad-based sustainable development, and essentially transform.

In the context of climate change, inclusion is particularly important (Shrestha et al. 2014). The urban poor, particularly in African cities, are significantly impacted by climate and disaster risk because of their exposure to multiple hazards and limited capacity to access resources and services (Revi et al. 2014; Adelekan et al. 2015; Ziervogel et al. 2017). As Fazey et al. (2018) outline, the community level is one where significant progress can be made on building resilience to cope with a 1.5 deg world (Fazey et al. 2018). Despite the growth of urban climate change policies and focus on climate change adaptation and governance (Bulkeley 2010; Aylett 2013; Heinrichs et al. 2013; Leck and Roberts 2015), insufficient attention has been placed on how local government might shift towards pro-poor planning that reduces climate risk in the context of upgrading informal settlements and supporting the informal economy (Bousquet et al. 2016; Amundsen et al. 2018). Regulations and plans designed for the formal economy and formal settlements need to be challenged, and made locally relevant, given 61% of urban African residents currently live in slums and rely heavily on the informal economy (Pieterse et al. 2015). In the context of increasing climate risk and the need for urban transformation, stronger relations between local government and the urban poor are needed to shape this rethinking and change (Few et al. 2007; Collins and Ison 2009).

Drawing on a number of cases from across Africa, Mapfumo et al. (2017) suggest the need to move from a focus on transformation outcomes to the processes through which transformational change is achieved. Underpinning this is the need to understand and support the development of transformative capacity (Wolfram 2016). In the context of cities, this capacity is needed across scales, from the individual and household level to the organizational level including local government and the private sector. A growing focus on cross-scalar adaptation governance has emphasized the importance of understanding relations across scales (Betsill and Bulkeley 2006; Pahl-Wostl 2009; Leck and Simon 2013; Termeer et al. 2016). Although there are examples of community-based adaptation and local groups taking action, there has been limited focus on the cross-scalar governance context of these responses (Moser et al. 2010; Dodman and Kiluma 2011). There are examples of social movements, such as Slum/Shack Dwellers International (SDI), that have found ways to mobilize federations of urban poor groups to work alongside NGOs and city governments, although state responses to this have been mixed (Mitlin 2008; McGranahan et al. 2016). Lessons should be learned from this and other fields, including disaster risk reduction, to explore how local governments might engage directly with citizens to unpack and collaborate around adapting to climate variability (Wamsler 2016), particularly with the urban poor (Archer et al. 2014; Chu et al. 2016; Fraser 2017). Contributing to this gap, the aim of this paper is to explore how transformative capacity for urban climate adaptation can be built through a shift to inclusive governance between local government and the urban poor.

Rather than looking at the transformation of the city system, this paper draws on two cases from the Western Cape, South Africa, to identify inflection points where there has been a shift in planning and practice that moves towards climate adaptation and transformation. This responds to Biesbroek et al.’s (2015) call for researchers to open up the ‘black box’ of climate adaptation implementation, to understand the inherently complex, socio-ecological and socio-technical system in which adaptation occurs. Transformation within the space of inequality and informality can serve as a litmus test. If the nature of how the poor interface with local government can shift, then it holds promise for better off urban residents as well. Interrogating these experiments can help to identify opportunities, as well as potential challenges, with these emergent approaches to reducing climate risk and strengthening transformative capacity. This paper starts by unpacking the framework used to explore transformative capacity, with a focus on inclusive governance. Contributing to the gap of practice-relevant research, it then presents two cases from South Africa where the urban poor have engaged with local government on climate risk and social change. The discussion goes on to explore the importance of participation, sustained intermediaries and diverse governance modes as ways to build transformative capacity. In conclusion, the paper reflects on how local government and the urban poor might engage, in theory and practice, to build transformative capacity that enables inclusive governance.

## Unpacking urban transformative capacity

With the growing focus on transformation, there has been an associated interest in the capacity needed to perform radical system change across various disciplines (Park et al. 2012; Wilson et al. 2013; Ziervogel et al. 2016). Within the fields of socio-technical transitions and climate change adaptation, there has been an interest in the capacity needed to accelerate social change (Gupta et al. 2010; Engle 2011; Wolfram 2016). Emerging from socio-ecological systems theory, the concept of adaptive capacity has been well established with a recent focus on transformative capacity that has been used to understand the capacity to transform social-ecological systems’ trajectories towards ecosystem stewardship at the landscape scale (Carpenter and Brock 2008; Olsson et al. 2010). Through an urban lens, Ernstson et al. (2010) stress the importance of transformative capacity when transitioning to a more preferable regime, emphasizing the role that social networks and innovation play in sustaining ecosystem services and navigating these urban transitions.

Transformative capacity can be defined in different ways because of the disciplinary plurality on which it draws (e.g. see Wilson et al. 2013; Wolfram 2016; Newton et al. 2017). Ziervogel et al. (2016, p. 955), building on the adaptive capacity and organisational development literature, define transformative capacity as “the capacity of individuals and organisations to be able to both transform themselves and their society in a deliberate, conscious way. This includes the capacity to imagine, enact, and sustain a transformed world and a way of life that is in balance with the carrying capacity of our earth, and where all life flourishes”. Focusing specifically on urban transformative capacity, Wolfram (2016, p. 126) defines it as “the collective ability of the stakeholders involved in urban development to conceive of, prepare for, initiate and perform path-deviant change towards sustainability within and across multiple complex systems that constitute the cities they relate to”.

**Fig. 1:**
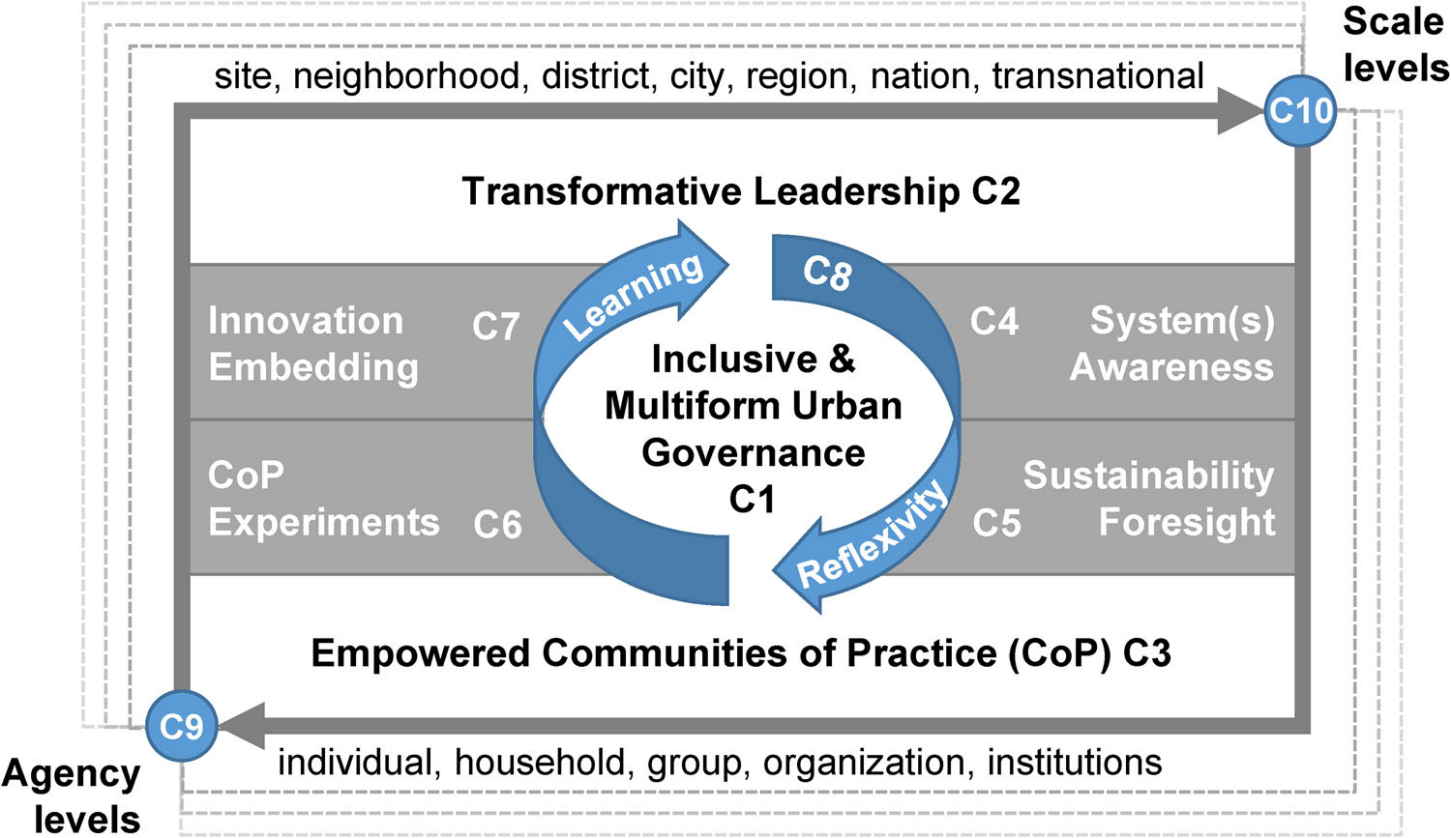
Overview—interdependent components of urban transformative capacity, centered on social learning and governance (cf. Wolfram 2016)

Given the radical and ambitious goal of urban transformation, it is not surprising that the transformative capacity needed to achieve this would include many components. Wolfram (2016), having identified a gap, draws on contributions from a range of disciplines, to put forward a framework for assessing urban transformative capacity for research and policy. In his methodic literature review, he identifies 60 factors, grouped into 10 interdependent key components of transformative capacity as shown in Fig. 1. For the purposes of this paper, component 1, of inclusive and multiform urban governance, put at the centre, is used as the lens of enquiry. This focus is justified by the argument in the introduction of the need for more inclusive urbanization and understanding the associated governance of how this might be achieved to contribute to transformative change. Component 1 is also an appropriate point of entry given the paper’s explicit focus on the interface between local government and the urban poor. Drawing on Wolfram (2016), the transformative capacity for shifting governance rests on three subcomponents as outlined in Table 1, namely (a) wide participation and active inclusion of stakeholders from all sectors, (b) diversity of governance modes and actor networks (de -/centralized, formal/informal, multi-level, etc.), and (c) sustained and effective intermediary organizations and individuals between sectors and domains (hybridization).

**Table 1:** Factors contributing to inclusive and multiform urban governance, as a component of transformative capacity ( *Source* adapted from Wolfram 2016)

Component 1: Inclusive and multiform urban governance	Key references
*Participation and inclusiveness* Citizens and civil society organizations, as well as private businesses and their representations, participate directly in the deliberation of actions with state actors (government, administration). Formerly excluded stakeholders are involved actively and supported to enable their contribution.	Avelino and Wittmayer (2015), Innes and Booher (2003), Pahl-Wostl (2009), Romero-Lankao et al. (2018)
*Sustained intermediaries and hybridization* There are intermediaries positioned between societal stakeholders that bridge relevant gaps amid sectors (public, private and civil society), action domains (e.g. energy/transport/land use), and/or spatial scales. Intermediaries have a stable financial and organizational basis. There are key individuals acting as boundary spanners or knowledge brokers between sectors, action domains and scales. Intermediaries effectively align different actor interests and help to create a shared discourse.	Avelino and Wittmayer (2015), Hamann and April (2013), Hodson and Marvin (2010)
*Diverse governance modes and network forms* There is diversity of formal and informal actor networks and governance modes. There is diversity of centralized and decentralized actor networks and governance modes (top-down/bottom-up; hierarchy/market/negotiation). Governance helps to build social and political capital Governance addresses multi-level and cross-scale implications.	Innes and Booher (2003), Pahl-Wostl (2009), van Buuren et al. (2015)

## Materials and methods

Two cases, both urban sites in the Western Cape, South Africa, are used in this paper to explore adaptation planning and implementation in practice. Using information-oriented selection, the cases were chosen based on both exhibiting direct relationships between local government and the urban poor (Flyvberg 2011). This critical selection was based on the evidence of positive change for the urban poor groups related to environmental and/or social risk that emerged from the cross-scalar relations.

The first case study, based in Green Park informal settlement, Cape Town, draws on empirical research undertaken between 2015 and 2017 to understand the implementation of gravel platforms by the City of Cape Town to reduce flood risk. Interviewees were purposively selected to represent various institutions and non-governmental organizations (NGOs) and different bureaucratic and political roles within government. The nineteen interviews were taped and transcribed with the consent of the participants. Field observations and informal non-recorded conversations with residents living in the settlement also contributed to the empirical foundation for this case.

The second case, in Piketberg, in the Bergrivier Municipality, draws on work from the Fostering Local Wellbeing (FLOW) programme. The aim of the FLOW programme was to “contribute towards fostering well-being in two towns in South Africa through developing adaptive and transformative capacity in the face of climate change and increasing economic inequality, particularly amongst the youth” (African Climate and Development Initiative 2014). The project took a transdisciplinary approach, where academics, practitioners, municipal officials and a group of citizens worked collaboratively to implement a range of interventions aimed at building transformative capacity for unemployed youth and local government (Ziervogel et al. 2016). The data in this case draw on a range of material collected during the programme, including interviews with officials, the FLOW coordinator, ambassadors and the project team members. In addition, a rich repository of data was drawn on from the ambassadors who frequently submitted responses to questions throughout the project. The case also draws on a number of workshops held during the course of the project and participant observation, as the author was part of the project conceptualization and implementation.

The lens of transformative capacity was used to guide the thematic analysis of the data from both cases (Braun and Clarke 2006), focusing on the extent of inclusive governance, and the themes of participation, intermediaries and diverse governance modes. Particular attention was paid to understanding the process of implementing responses as well as how relationships between local government and the urban poor have changed.

### Study area

To place the case studies in context, it is worth noting that South African cities are some of most unequal in the world. Much of this is due to the historic socio-spatial legacy of apartheid that saw townships pushed to the outskirts of urban areas, remaining poverty traps today (McGranahan et al. 2016). Rapid immigration to cities from rural areas and other African countries are increasing the challenge of securing houses, services and jobs, particularly for the urban poor, but also creating opportunities and innovation (Pieterse 2017). Informal settlements, where people build their own houses from corrugated iron and scraps, are usually on marginal land, exposing citizens to high levels of environmental risk such as flooding. Ecosystem services are also pushed to their limits due to pollution and overuse of resources, due to a lack of formal resources and service availability (Seeliger and Turok 2013). In some of the smaller towns, the youth want to move to the cities, where they see potential for better jobs, education and a better life (Ziervogel et al. 2016). Although the urban poor are exposed to high environmental risk and climate variability, they often have a strong vision of the future they desire and what suitable adaptation responses would be.

The Western Cape, where the case studies are located, is better off than many of the other eight provinces in South Africa. Western Cape citizens have benefited the most over the last decade from free basic water, electricity and sanitation and sewerage services (StatsSA 2016). Cape Town is the biggest city in the province, with around 4 million people. It has large informal settlements where residents often travel far to get work, if they are able to find it. Piketberg, the administrative centre of the Bergrivier Municipality, about an hour and a half from Cape Town, is a typical small town, with strong links to the adjacent rural areas where farming dominates the local economy. It has few shacks, but poverty levels are high. Increasingly there is a strain on agricultural jobs, impacting job opportunities in the area.

## Results

### Case study 1: Green Park gravel platforms

We have been promised for a long time, and we did not want only promises - so it was a rocky kind of relationship but it actually made a difference to where we are now. We are more kind of understanding the city now. We are understanding the dynamics and the challenges that they are faced with. The relationship is rocky because we are desperate of course - but things are normalizing after the city will agree to finance the electricity situation. (Green Park informal settlement leader, June 2016)

Green Park informal settlement is different to many other informal settlements. It is located inside a nature reserve and many of the shacks around the edge of the settlement have small gardens where people grow flowers and vegetables. There is frequent winter flooding on the low-lying land that borders the wetland area.

Because of the nature reserve status, the settlement land fell under the jurisdiction of the Western Cape Government and not the City of Cape Town. Despite this, the City wants to build formal low-income housing in part of the area. In order to proceed with services, the land had to be deproclaimed, so that the City could take ownership. This process, which started in 2005, was finalized in 2017.

**Fig. 2:**
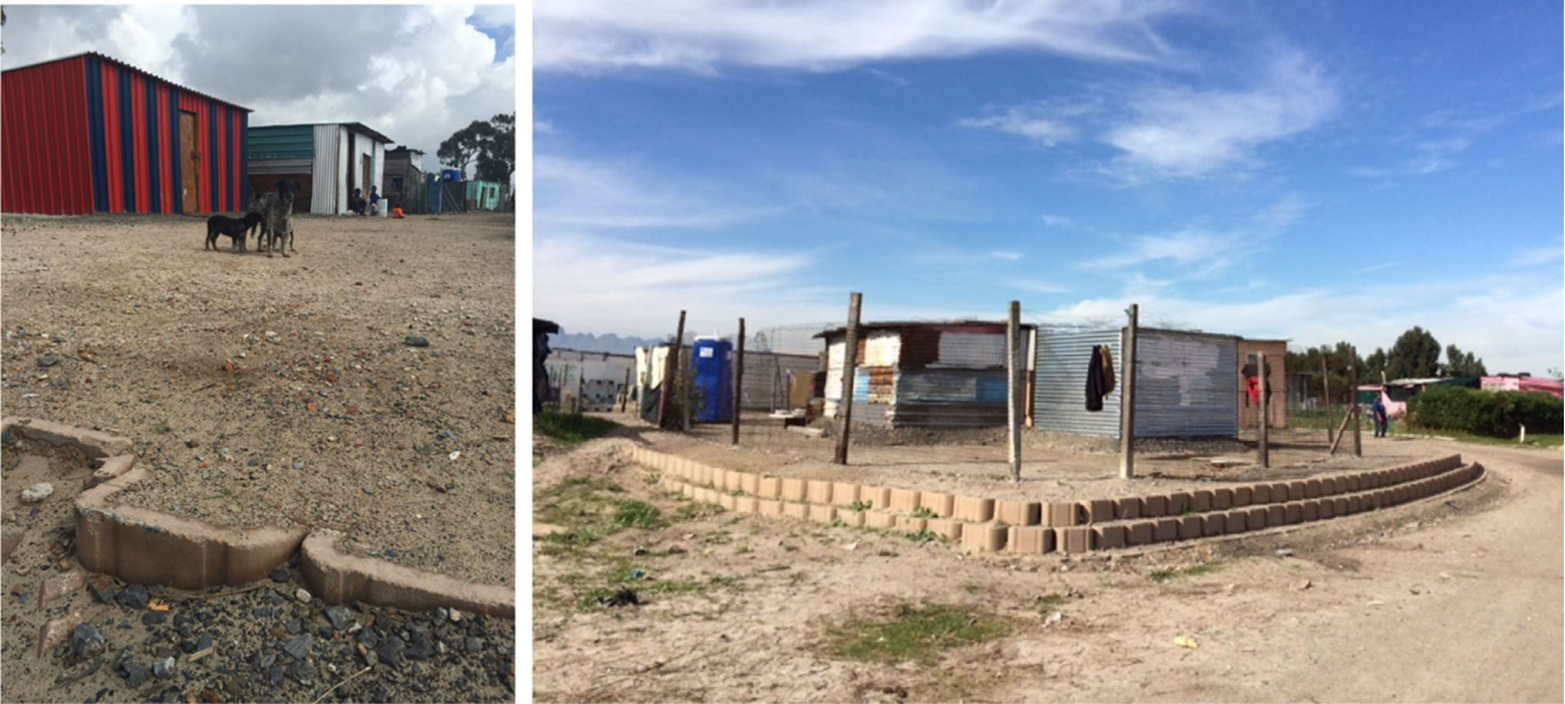
Examples of the gravel platforms that have been constructed in Green Park informal settlement

While waiting for authorization to provide formal housing, the City started to upgrade parts of the settlement. In 2015, three large gravel platforms were built. The large flat composite-gravel areas raised the ground level so that the relocated shacks no longer sit in the flooded wetland area (see Fig. 2) (Noorbuckus and Ziervogel 2018). Although the gravel platforms aimed to reduce flood risk, the settlement used their implementation to push for electricity, which could not be installed if there was flooding (Jordhus-Lier et al. 2018). After the platforms were built and flooding reduced, the City then installed electricity.

#### Moving towards inclusive governance

Although the outcome of the gravel platforms can be seen as incremental adaptation to climate risk, the governance process that led to the implementation of the platforms has elements of a transformative approach. The nature of engagement between the City of Cape Town officials and the settlement leader, Raymond, is an example of a shift in formal ways of governing to a more hybrid mode that draws on both formal and informal institutions.

The case shows how Raymond acted as an intermediary between his settlement and local government, while disrupting the formal planning processes. His leadership drew on his strong social networks and a history of being involved in the struggle against apartheid to mobilize action and draw attention to Green Park. Raymond and a group undertook a march to Parliament where they took a memorandum to the Mayor. Raymond was aware of the official protocol to speak to the Ward councillor first, who is expected to take issues up to higher levels. However, in an attempt to secure services, Raymond recognized the need to transgress the formal procedures, as he states below:

R: The project itself is long overdue. We are forced to speak to the relevant authorities, which is on top. This is out of desperateness. We are supposed to follow protocol: Council, then Ward councillor and moving up. However, there is not enough time. And also as far as the ward councillor is concerned, we would say as far as her being away, we had to find our own way.

I: OK, so what you did was to take “short cuts” not going through the normal channels?

R: Yes, that would be right. Ok, so let me put it this way… it was our initiative. You remember that I said that out of being desperate - we went out of our way to need a meeting with the Director of Human Settlements. To kind of make things happen. Because if we did not do that, we would not be where we are now. The platforms would not exist.”

The Mayor’s office put Raymond in contact with the Director of Urbanization in Human Settlements. She met with them and agreed to prioritize their settlement. After a MAYCO (mayoral committee) member visited the settlement, the Director gave permission to explore options for supporting the local requests. Although the residents were pushing their agenda of trying to secure electricity, the Director said she could help them sooner by addressing the flooding issue first.

Formal engagement from a wide range of government departments across spheres, from the city to provincial to national level, to address issues related to electricity, land ownership, formal housing and flood risk reduction leads to confusion over the status of planning and progress at different times (Jordhus-Lier et al. 2018). Often different departments’ information and updates did not align, with confusion over who was responsible for what and what processes needed to be followed. Raymond clearly expressed his frustration, saying that in 2006 they were told the area was going to be formally developed but in 2015 they were talking about interim solutions. Despite this, Raymond acted as an intermediary, aligned actor interests and created a shared discourse between officials and residents. Although there was concern about moving on to the platforms, Raymond encouraged households to do so in return for the promise of electricity, which was finally secured in late 2016. Through Raymond’s ability to read both the needs of the settlement residents as well as what the City could support, he was able to shift the trajectory of services in the settlement towards what residents wanted, rather than waiting for the City to decide on their priorities. This was achieved by drawing on diverse modes of governance including both formal and informal and top-down and bottom-up.

Although the case shows how an intermediary shifted the development trajectory of the settlement, broader participation and inclusion of diverse voices was lacking in the Green Park case. Once the City of Cape Town approved the platforms, a committee from the settlement was appointed, which allowed them to help steer the project and ensure local residents were part of the construction team. Unfortunately this did not provide the opportunity for a broader conceptualization and co-production of settlement plans, but rather relied on residents’ engagement with Raymond, as a leader. Building capacity for citizens to deliberate with the state actors could have produced more opportunities for transformative change.

Despite some challenges, this case highlights the importance of intermediaries in inclusive governance and the importance of navigating both the formal and informal institutions to find areas of overlapping goals between local government and the urban poor. The cross-scalar process enabled Raymond to bring his and some of the residents’ vision of securing housing and electricity to the fore. The implementation of gravel platforms to reduce flood risk was not part of this long-term vision but rather an intervention that reduced climate risk and was used as a step towards achieving their goal. This emphasizes the importance of contextualizing climate adaptation within the broader social development context, and as part of reducing structural vulnerability (Pelling 2011; Jordhus-Lier et al. 2018). The integration of local and city processes, and the role of a local leader were central to how the process unfolded. This case suggests that more emphasis should be placed on understanding how to support individual leaders, and integrate them in a larger structure, to support transformative capacity further.

### Case study 2: FLOW (Fostering local well-being) programme

This programme [FLOW] trained a group of youth in many aspects of Local Government Services and suddenly they started attending Municipal meetings with community leaders and provided us with a youth view that we value and listen to. The Council saw the big change in the ambassadors and renewed their commitment to youth development. (Municipal Manager, 2016)

Engagement in the Piketberg case started around their municipal climate adaptation plan in 2014 which led to the FLOW programme that worked with unemployed youth and the local municipality to explicitly build transformative capacity (www.flowafrica.org). The programme included three mutually reinforcing interventions—a youth leadership development programme with two cohorts of eight FLOW Ambassadors (FAs), the introduction of a local community currency (not discussed here) and local government support for strengthening engagement with civil society.

The FLOW coordinator, Ian, was central to liaising between the project team and the ambassadors. He also worked collaboratively with the practitioners and academics to develop activities and oversee the day-to-day programme. The youth leadership development component included activities ranging from resource flow mapping, enumerating a quantitative baseline survey with informal businesses to learning storytelling skills through video journalism. In order to build the agency and personal development of each ambassador, activities such as daily reflective practices and leading the group were included. Through different activities, including the survey, movies and participatory forums, the ambassadors interacted extensively with the municipality and local businesses.

The local government officials were directly involved in the project development and operation, which led to strong cross-scalar engagement. The municipal strategic manager, who drove the climate adaptation plan, was centrally involved in the initial stages of the programme but then left the municipality in 2016. The Local Economic Development manager was involved in day-to-day interactions with the FAs and frequently visited their office to get an update on their activities and share in the reflection space. The municipal manager was actively involved and central to decision making around how the project proceeded. Her leadership and enthusiasm helped to get buy-in at the highest level, both politically and administratively, which was central to FLOW’s success.

#### Moving towards inclusive governance

Although engagement in the region started around climate change adaptation, it became clear that the range of social, economic and ecological stresses faced by unemployed youth and other urban poor required a more holistic response than focusing solely on climate impacts. Although elements of specific capacity to respond to climate stress were included in the project, the focus shifted to strengthening generic capacity to respond to multiple stressors and transformative capacity to actively change current pathways (Eakin et al. 2014). This systemic understanding and vision was central to the success of the programme. As the municipal manager said,

It started as a climate change adaptation programme and grew into something totally unexpected through the interaction of research partners and practitioners in environmental management and their need to do something amazing with real impact on the ground. Genius!

The importance of understanding the socio-ecological system has been emphasized as central to supporting urban transformation (Ernstson et al. 2010). In order to do this, a central thread of the FA’s programme was to understand and make visible life-support systems. One way this was supported was by developing the ambassadors’ skills in mobile journalism. After intensive training, the ambassadors made many short movies featuring local entrepreneurs, local events, municipal and ecosystem services among others. In order to understand the municipal services related to water and waste, the ambassadors undertook a number of site visits. These included visits to the water extraction plant next to the Berg river, landfill sites and an overnight expedition to the Grootwinterhoek mountains to gain insight into where the town water comes from and how it is part of the urban water cycle. During a visit to the water treatment works, a group of FAs put together a two-minute video about how waste water is treated.

These videos not only helped to give the ambassadors a better understanding of life-support systems, but were used by the municipality to provide a different perspective on municipal services. At the IDP (Integrated Development Programme) forum in 2016, the video of the water treatment works was shown to participants including municipal officials, politicians, business representatives, community organization representatives and many others who were invited to give input to the municipal plans for the year. The screening of the short video was an important milestone. The municipality was happy to have their services shown, as people often grapple with what the municipality does because many of the services go unnoticed. The ambassadors were also delighted to show their movie to a large audience, and in doing so provided a local perspective on environmental issues at the municipal level. This talks to the shift towards inclusiveness, through valuing the knowledge and contribution of a group of actors whose voice has not been heard much in the past, which in this case was a group of urban youth.

Another aspect of transformation could be seen in how the project was funded. A large component of the project was funded through the University of Cape Town with an international grant. Although this supported most of the work, it could not support payment of the ambassadors’ time. So in the first phase, the Bergrivier municipality managed to secure funding for the stipends of the FAs through the National EPWP (Expanded Public Works Programme). As the municipal manager said, “At the beginning we started it as an EPWP project, and it didn’t fit there but we hammered it to fit there because we didn’t have another way to get money for it, so we actually put a round circle in a square box that first year just to make it work”. For the second year the municipal manager requested the Municipal budget steering committee to take the money for stipends direct from the municipal budget, which they did despite limited resources. This helped to embed the project explicitly in the municipality’s activities. Although the university engagement in the project ended at the end of 2016, the municipality secured funds for the FLOW coordinator and two of the old ambassadors to train a new cohort of ambassadors in 2017. The fact that the project continued despite the withdrawal of the main funding from the University points to its sustainability and the importance placed on it by both the municipality and the youth.

Central to the success of the programme was the FLOW coordinator, Ian, who was appointed from the area. One of the project members commented that Ian was “exactly the right jockey because he is an entrepreneur and a farmer”. His role as a skilled intermediary supported the interaction between the municipality, the ambassadors and with the wider networks that the ambassadors were part of, thereby enabling a new form of cross-scalar governance.

**Fig. 3:**
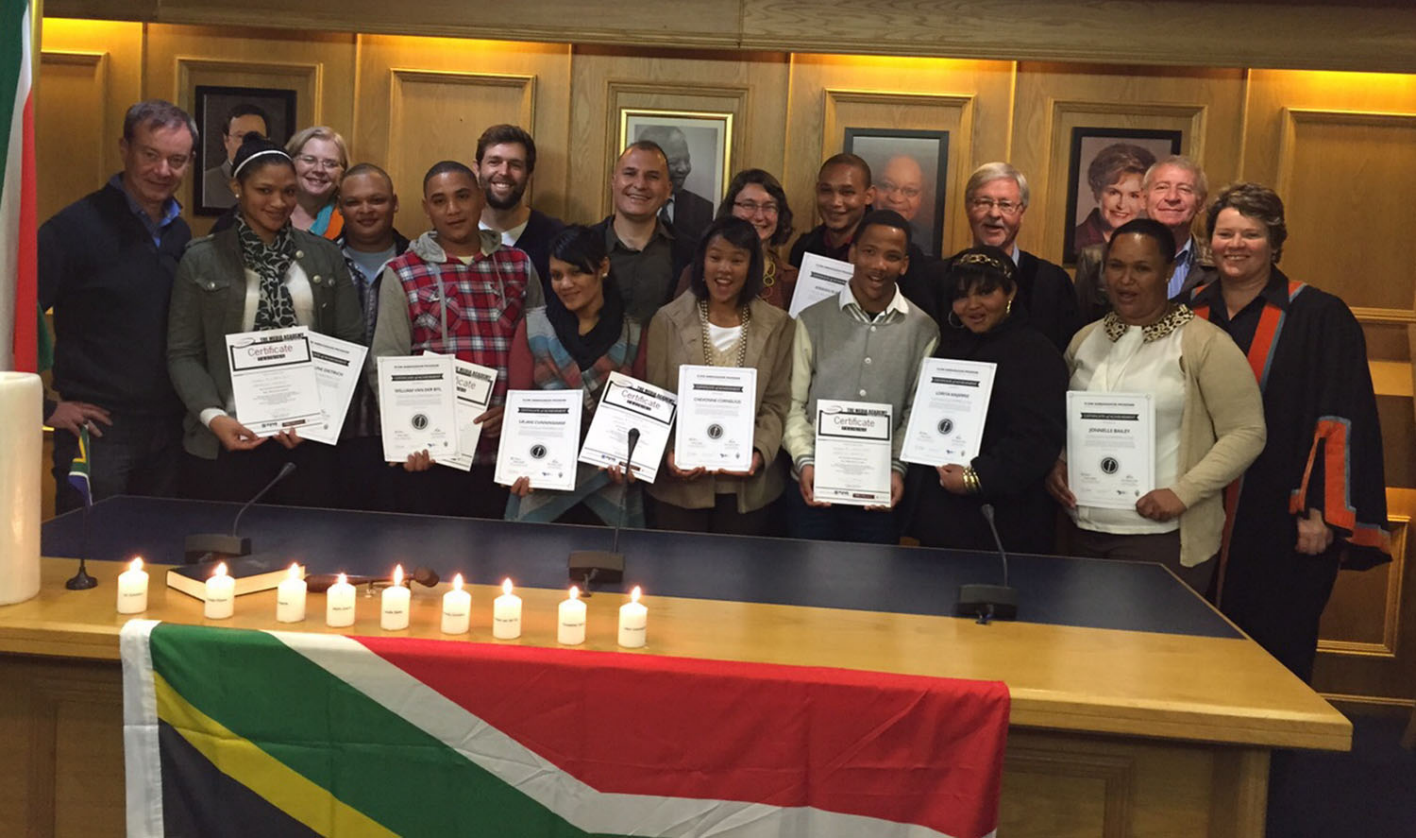
The FLOW ambassadors (in the front) receiving their certificates after a ceremony in the municipal chambers

Through the FA leadership programme, there was significant personal growth in the youth that helped to support broader participation in municipal processes. The FAs attended various meetings hosted by the municipality and provided input into various processes, such as the IDP, showing how the voice of the urban poor started to be recognized in these public forums. This speaks to how the governance structures started to shift and provide space for more diverse voices. As the Municipal Manager reflected, “they are not afraid to lift their hand and say ‘hear me’ and then 60 people will quiet down and listen to them. We never had youth like that before”. The Municipal Manager was equally moved by the closing ceremony (shown in Fig. 3) when the FAs graduated,

I think the FLOW programme gave the FLOW Ambassadors a sense of achievement and hope. At the certification ceremony every one of them had a five minute speech in front of the whole room. They stood up, they were upright, they looked the people in the eye, they had charisma. Most of them referred to the fact that they now know that they can become anything they want and that they can make a success of a career. They were just so changed, I couldn’t believe my eyes. That’s over and above the other skills that they gained.

The ambassadors’ comments on what they got out of FLOW show how their personal perspectives shifted, which O’Brien (2018) argues is such an important component of transformation. The ambassadors felt heard by the municipality and project team and started to see themselves and their town as part of a bigger system. As one of them said, “Now I can speak to all types of people, from different walks of life. Down-to-earth people and high brow professionals”. Most FAs testified at the graduation ceremony that before the programme they did not know anything about how the municipality secured services such as drinking water, electricity, refuse removal and sewerage, but now they did. The FLOW coordinator commented more specifically on how the programme helped him to understand the surrounding natural resources, saying, “I learned that we are so lucky to have so many natural resources here in our area, and that we’re so dependent on them, but also that we’re very vulnerable. We have to look after these, because without them we won’t be able to live here”.

The outcomes of the project speak to the importance of strengthening individual agency and supporting changes to the governance system as an important first step in strengthening inclusive governance before focusing explicitly on reducing environmental risk. As one of the FLOW team commented, “What FLOW has achieved is just the start of an imagined future in which planning will be participatory and owned by the community”.

## Discussion

### Building transformative capacity through inclusive governance

As Carter et al. (2015, p. 57) suggest “there is a need to move beyond sustainable urban visions towards the grounded creation of new interdisciplinary networks, adaptive capacities and collaborative practices, assembled to respond to the adaptation imperative at the urban scale”. This is new territory, grappling with the anthropocene, uncertainty, greater social inclusion and all its challenges. One starting point is to focus on how local government and the urban poor have and could change their relationships and interaction to reduce climate risk and lead to broader transformation. Drawing on a practice-oriented account of two urban cases in South Africa, this paper puts forward three points on how inclusive governance, at the core of transformative capacity, could be strengthened, while recognizing links to the other components of transformative capacity (Wolfram 2016).

First, to reduce climate risk for the urban poor their everyday reality and existing governance modes should be the entry point to identify priorities for transformative change. If officials could draw on diverse understandings of the system, more holistic opportunities for reducing root causes of vulnerability, which need to underpin transformation, could be developed (Pelling et al. 2015). In the FLOW project, the initial focus on climate adaptation shifted to focus more on root causes linked to poverty and the informal economy, as well as social networks and agency. As the project progressed, the focus shifted back to understanding life-support systems and climate risk, as by then the ambassadors could better appreciate the importance of reducing environmental risk. Similarly in the Green Park case, the flood platforms were used to secure services such as electricity that were a local priority. Situating adaptation responses in the everyday reality and politics of the urban poor gives more space to identifying transformative approaches that might both reduce climate risk and structural vulnerability (Fraser 2017). The realities of urban daily life and “cityness” are often overlooked and displaced by rational policy fixes whose impact is then limited (Pieterse 2010). As Menkhaus (2007, p. 75) outlines, local efforts of governance “reinforce the obvious but often overlooked observation that local communities are not passive in the face of state failure and insecurity, but instead adapt in a variety of ways to minimize risk”. These local governance explorations, which include informality, everyday practices and institutions (both formal and informal), need to be embraced and interrogated for how they could be leveraged to lead to transformation (Fraser 2017). It is not to say they are the answer, but should be seen as an important entry point.

Moving from the theory of integrating local perspectives and experiences into planning and practice is not easy (Mitlin 2008). Left to their own devices, local authorities are unlikely to plan for inclusive governance (McGranahan et al. 2016). Pressure from below is needed to shift authorities’ responses away from centralized control that seldom supports democratic practice, to more inclusive plans and policies. More support is therefore needed for experiments that support the aspirations that the urban poor have around adaptation and transformation (Fazey et al. 2018). The two cases in this paper looked explicitly at examples of inclusive governance where there was not an organized social movement or grassroots organization, which have been shown to hold potential for inclusive governance (Mitlin 2008). Rather, it asked how the urban poor might engage in inclusive governance directly with local government, in one case through an emergent process driven by an informal settlement leader and the other through a transdisciplinary project. In both cases, the perspectives of the urban poor on the nature of the local challenges were given attention. In neither case was this done through traditional participatory processes such as workshops and state-driven consultation. Rather, relationships were built through formal and informal channels. In the FLOW programme, the explicit attention in the ambassador’s leadership programme to developing agency of a group of youth was central to this. The diverse activities and opportunities contributed to local empowerment and provided opportunities to engage directly with local authorities. This interaction across scales can be a powerful force in contributing to transformation (Pelling et al. 2015).

Second, sustained intermediaries, who are urban poor themselves, can play an important leadership role in driving inclusive governance and therefore need to be better supported. Because intermediaries link distinct groups, they can contribute specifically to cross-scalar governance. Within the climate change literature, the focus on leadership has been on formal adaptation planning and government activities and less on leadership from within civil society (Moser and Ekstrom 2010; Measham et al. 2011; Pasquini et al. 2015). Chu et al. (2016) highlight youth leadership as an important enabler in reviving traditional agricultural practices in Quito. Raymond, from Green Park, and Ian, from the FLOW programme, both live in low-income areas and were able to work closely with others from their community as well as with local government officials, and so demonstrated idiosyncratic leadership. Their personal relationships with local government officials provided opportunities for influencing both formal and informal planning and decision-making processes (Leck and Roberts 2015). The sustained intermediary is central to building inclusive cross-scalar governance. These intermediaries can also help to explore alternative system configurations that can help to build transformative trajectories (Pahl-Wostl 2009).

Third and last, local governments need to draw on diverse modes of governance to engage more fully with the urban poor. Contestation and authentic deliberation around planning and adaptation needs to be embraced, particularly in highly unequal societies (Pasquini et al. 2013; Hamann and April 2013). As the FLOW project demonstrated, transformative capacity was built through a transdisciplinary process that supported experimentation across scales from local government to citizens. In Green Park, a settlement leader drew on informal social networks to initiate a process of change. He also subverted formal planning processes to meet the development goals of residents as well as reducing their climate risk. Governance modes that shift to address power inequalities, build social cohesion and work across scales and diverse actor groups need to be supported (Pelling et al. 2015; Ziervogel et al. 2016).

## Conclusion

Traditional modes of planning, existing competencies and sectoral approaches in cities are insufficient for achieving climate adaptation, urban resilience and risk reduction (Adelekan et al. 2015; Barnett and Parnell 2018; Solecki et al. 2018). Although large scale change is needed, it is just as important to acknowledge that incremental adjustments can also move a system towards transformation (Pelling et al. 2015). Talking about climate governance, Frohlich and Kneiling (2013) call for a “broad variety of approaches and solutions” to address the multiplicity of interests. The two cases presented here contribute to this pot of examples on inclusive governance in the urban context. This could be expanded in future research to understand how other components of transformative capacity might enable transformative change for the urban poor. Given the high levels of informality in African cities, and increasingly complex governance settings, further research is needed across different contexts and scales.

The dominance of technical, managerial approaches and an economic rationale for urban development that have characterized local authorities has tended to move adaptation planning away from transformative orientations to support the status quo (Castán Broto 2017). City government policies and planning tools have tended to exclude low-income residents (McGranahan et al. 2016). Changing these patterns and practices is urgent but challenging (Romero-Lankao et al. 2018). Recognizing that change is needed is the first step. This paper argues that one place to begin is by actively building relationships between local government and the urban poor. To do this, partnerships, transdisciplinary approaches and experimentation are needed to support deliberation and social learning that can open up these new possibilities for transformation (Hordijk et al. 2014). This will require new types of training for government officials, activists and researchers, new types of funding and new modes of governance to enable place-based stakeholders to have more influence over changes in their space.
